# Gastrointestinal stromal tumours of the stomach: Cytological and immunocytochemical diagnostic features of two cases diagnosed by endoscopic ultrasound-guided fine needle aspiration

**DOI:** 10.3892/ol.2013.1296

**Published:** 2013-04-10

**Authors:** P. TODARO, S.F. CRINÒ, S. PALLIO, C. FAZZARI, P. CONSOLO, G. TUCCARI

**Affiliations:** 1 Department of Human Pathology, University of Messina, University-Hospital Health Network ‘Polyclinic G. Martino’, Messina I-98125, Italy; 2 Digestive Endoscopy Unit, University of Messina, University-Hospital Health Network ‘Polyclinic G. Martino’, Messina I-98125, Italy

**Keywords:** gastrointestinal stromal tumours, cytopathology, immunocytochemistry, cell block, differential diagnosis

## Abstract

The present study reports the diagnostic utility of endoscopic ultrasound-guided fine needle aspiration (EUS-FNAC) in two patients affected by gastrointestinal stromal tumours (GISTs) of the stomach. Clinically, the patients demonstrated skin pallor, melena, gastric discomfort and pain that had lasted three days or weeks. The cytological findings are discussed; these were strongly supported by immunocytochemical procedures that were performed on cell blocks and further confirmed following post-surgical histopathological examination. The crucial aim of GIST management is to determine a correct diagnosis in early-phase disease in order to realize an adequate curative surgical resection before the tumour becomes unresectable or metastatic. Moreover, a correct pre-surgical differential diagnosis of GISTs from other mesenchymal neoplasms may be easily made by EUS-FNAC, supported by cytological and immunocytochemical features.

## Introduction

Gastrointestinal stromal tumours (GISTs) are uncommon mesenchymal neoplasias of the gastrointestinal tract that may occur between the oesophagus and anus, and even in the omentum and mesentery (1–7). GISTs are found infrequently in adults prior to the age of 40, generally presenting with a peak incidence during the fifth and sixth decades and without significant gender differences (2,3,8). The histogenesis of these tumours has been attributed to the interstitial cells of Cajal, which are referred to as the pacemaker cells of the gastrointestinal tract (3–6) and which are immunohistochemically positive for CD117 (5–7,9).

The clinical presentation of a GIST is largely dependent on the site of occurrence, as well as the size of the tumours, although the clinical signs and symptoms, including nausea, abdominal pain, weight loss, anaemia or melena are non-specific and therefore not useful for the diagnosis (3–7). However, patients may also present with signs of obstruction, perforation, palpable masses and peritoneal seeding (2,6,7,10,11).

A large series of GIST cases revealed that these tumours have a broad spectrum of clinical behavior at all sites of occurrence. However, they are considered to be potentially malignant (5,12–14) and therefore require a multidisciplinary approach to optimise the management of patients. An accurate and early diagnosis of these rare tumours affects the treatment, primarily allowing the chance of an optimal surgical resection, which may reduce the number of unresectable or metastatic GIST cases. However, imatinib mesylate is now regarded as the revolutionary standard care in the first-line treatment of advanced GISTs (5,15–17).

The present study reports two cases of gastric GISTs that occurred as submucosal or intramural nodules. The diagnosis of a GIST was achieved by endoscopic ultrasound-guided fine-needle aspiration cytology (EUS-FNAC) and immunocytochemistry, which was further confirmed by post-surgical examination. Written informed consent was obtained from both patients; the original corrresponding declarations were available at the Department of Human Pathology, University of Messina, Italy.

## Case reports

### Case 1

**Figure 1:**
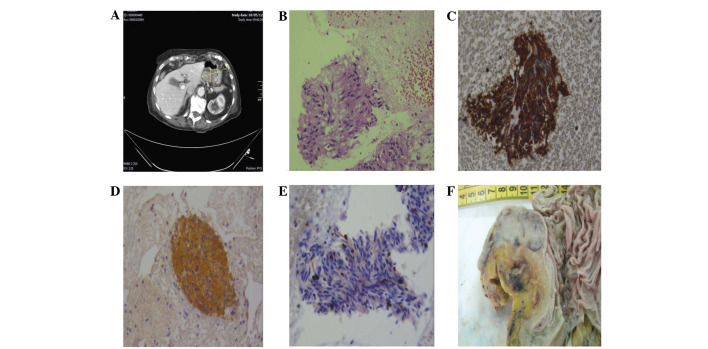
Case 1. Coronal CT scan showing a hypodense mass with not homogeneous contrast enhancement developing from the small gastric curve and causing (A) a partial reduction of the gastric lumen. (B) Cytological smears exhibiting aggregates of spindle cell elements with elongated nuclei (haematoxylin-eosin, ×160); the same elements were intensely immunoreactive for (C) vimentin (immunoperoxidase, ×200) and (D) CD117 (immunoperoxidase, ×120), showing only a sporadic nuclear immunopositivity for (E) Ki67 (immunoperoxidase, ×200). (F). The surgical specimen revealed the gastric sub-mucosal localisation of the GIST. GIST, gastrointestinal stromal tumour; CT, computed tomography.

An 88-year-old female presented with melena that had lasted 3 days. A general examination revealed a moderate pallor without weight loss or pain and no evidence of free fluid in the abdomen. Computed tomography (CT) revealed a 40-mm oval lesion with defined margins measuring 56.4×33.5 mm. The lesion was localised in the submucosal layer of the gastric wall between the corpus and the antrum, along the small gastric curvature (Fig 1A). No lesions were evident in the pancreas, biliary tree, duodenum and lymph nodes. EUS-FNAC was performed using a convex array echoendoscope (EG 3870 UTK; Pentax, Tokyo, Japan) and by making two passes with a 25 gauge needle. The specimens were stained with haematoxylin and eosin, processed by an in-room cytopathologist and then immediately examined for adequate cellularity. A second slide was fixed in 95% ethanol and Papanicolaou’s stain was applied. Any excessive material, including the needle and syringe utilized in the procedure, was rinsed in 10 ml 50% ethanol in a specimen container. All content was centrifuged in a 10 ml disposable centrifuge tube at 5,017 × g for 6 min to create 1 or 2 pellets. The supernatant fluid was decanted and the pelleted material was immediately fixed in a freshly prepared solution of 4% neutral buffered formalin for 45 min. The cell pellets were then placed in a cassette and stored in 80% ethanol until they were ready for processing in an automatic tissue processor (Leica TP1020; Leica Biosystems, Ltd., Mannheim, Germany). The cell blocks that were obtained were embedded in paraffin at 56°C and successive 3-*μ*m thick sections were cut and routinely stained by haematoxylin and eosin. Parallel serial sections of the same thickness were mounted onto silane-coated glasses and submitted for immunohistochemical procedures, as previously described (18–20).

### Case 2

**Figure 2:**
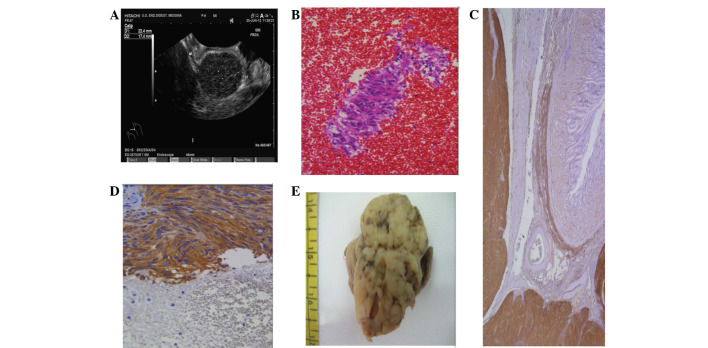
Case 2. (A) EUS scanning revealed a 22.4×17.4-mm, hypoechoic, well-delimited lesion, originating from the muscle layer. (B) The cytology of the lesion was strongly suggestive of a GIST, being formed of clusters of spindle cells (immunoperoxidase staining; magnification, ×200). (C) Upon histological examination, a diffuse cytoplasmic CD117 immunoreactivity was found in the proliferative spindle cell elements of the gastric wall (immunoperoxidase staining; magnification, ×120). (D) The clusters of spindle cells were reactive for CD117, but also for CD34 (immunoperoxidase staining; magnification, ×160). (E) The cut surface of the surgical specimen showed a white-greyish nodular feature. EUS, endoscopic ultrasound; GIST, gastrointestinal stromal tumour.

A 76-year-old male presented to the Surgery Department, University-Hospital Health Network ‘Polyclinic G. Martino’, with gastric discomfort and pain in the mesogastric region that had lasted three weeks. During a general examination, the pain increased with palpation and a pale skin tone was noted. Ultrasonography of the abdomen revealed a 22.4×17.4-mm hypoechoic round lesion with a well-defined margin. The lesion was localized in the superficial muscular layer of the gastric corpus, between the posterior wall and the large gastric curvature (Fig. 2A). No lesions were evident elsewhere in the abdominal organs. EUS-FNAC was performed with the same procedures that had been utilized in case 1; adequate cellular smears and one cell block were obtained.

Following the FNAC procedures, the two patients were observed for a period of 48 h for any procedure-related complications.

### Cytological and immunocytochemical findings

The smears from the two cases exhibited haemorrhagic backgrounds with a well-represented cellularity. They were organized in cohesive groups, arranged in three-dimensional clusters or as single cells (Figs. 1B and 2B). The elements were spindle-shaped with scant, lightly eosinophilic cytoplasm and elongated/oval plump nuclei. The chromatin was clumped with indistinct nucleoli and mild pleomorphisms (Figs. 1B and 2B). No mitoses were identified. In case 2, the spindle cell elements occasionally exhibited paranuclear vacuoles with an epithelioid feature. A presumptive diagnosis of gastric GIST was made for the two cases.

The cell blocks documented an equivalent morphology characterized by monotonous sheets and groups of spindle-shaped cells with oval nuclei and well-defined cellular borders. Mitotic activity was virtually absent. Immunohistochemical procedures were carried out on the 3-*μ*m serial sections, utilizing the following commercially obtained antisera from DakoCytomation (Copenhaghen, Denmark): CD117 [(working dilution) w.d., 1:150], CD34 (w.d., 1:200), smooth muscle actin (SMA; w.d., 1:200), vimentin (w.d., 1:250), S-100 (w.d., 1:400), desmin (w.d., 1:250), glial fibrillary acidic protein (GFAP; w.d., 1:300) neurofilaments (NF; w.d., 1:300) and Ki67 (MIB-1; w.d., 1:50). In cases 1 and 2, a strong and diffuse cytoplasmic immunostaining was encountered for vimentin (Fig. 1C), CD117 (Fig. 1D) and CD34 (Fig. 2C). The majority of the spindle-shaped clusters also exhibited immunoexpression for SMA. No immunostaining was recorded for desmin, S100, GFAP or NF. The growth fraction, determined by Ki67 as the MIB-1 labeling-index, was extremely low, showing <5% of positively-labelled nuclei (Fig. 1E).

### Gross and microscopic examination

The patients of cases 1 and 2 underwent surgical laparotomy and were alive and well at 12 and 8 months post-surgery, respectively. The resected tumours were sent for histological analysis. In case 1, a gross examination revealed a white-greyish nodular growth measuring 40×21 mm, situated below the mucosal surface of the stomach (Fig. 1F), while case 2 showed an intraparietal whitish nodular mass with a maximum diameter of 23 mm (Fig. 2D). Upon microscopic examination, the two lesions were observed to be formed from uniform sheets and interlacing fascicles of spindle-shaped cells, exhibiting elongated or oval nuclei, without atypia and with occasional mitoses. The immunohistochemical analysis documented an intense cytoplasmic positivity for CD117 (Fig. 2E), CD34, SMA and vimentin, while desmin, S100, GFAP and NF were largely unreactive. Immunoreactive nuclei for Ki67 were encountered in <5% of the proliferating spindle-shaped elements.

### Discussion

EUS-FNAC and endoscopic ultrasound-guided tru-cut biopsy (EUS-TCB) have been proven to be of significant value in the diagnostic evaluation of benign and malignant diseases, as well as for the staging of malignant tumours of the gastrointestinal tract and adjacent organs (21–23). The diagnostic yield of EUS-FNAC partially depends on the site, size and characteristics of the target tissues as well as certain technical/procedural factors. However, it is mainly dependent on the expertise, training and interaction between the endosonographer and cytopathologist. EUS-TCB utilizes a stiffer device that appears to be marginally more difficult to use than the standard FNAC. Currently, there are no accepted standards for when EUS-TCB should be used to improve diagnostic accuracy (24). In the present study, adequate cellular smears and corresponding cell blocks were obtained using the EUS-FNAC approach to gastric GISTs. Subsequently, spindle shaped cells with scant cytoplasm and elongated/oval nuclei were identified, which were strongly suggestive for a cytological diagnosis of a GIST. Moreover, a confirmatory immunocytochemical investigation was performed on the available material, with evidence of CD117, CD34, vimentin and SMA immunostaining in the considered cellular proliferations. These morphological data have been verified by histology and immunohistochemistry following a post-surgical examination of the resected tumours. Thus, an early, accurate diagnosis ensured the use of an appropriate therapy for the patients, and the two cases should be included in the suggested algorithm (CD117, CD34, vimentin and SMA) for GISTs, as has previously been described (5,14).

Another notable point from the present study is the pre-surgical opportunity to perform a correct differential diagnosis of gastric GISTs from other mesenchymal neoplasias, including leiomyoma, schwannoma and solitary fibrous tumours, or alternatively, metastatic diseases, such as spindle cell amelanotic melanoma or carcinoma. The additional value that immunohistochemistry may provide as a diagnostic confirmatory procedure, together with the specific cytological findings, should be considered either in smears or in cell blocks. Leiomyomas are strongly positive for desmin and SMA, but negative for CD117 and CD34, while schwannomas show positivity for S100 protein, with a lack of CD117 and CD34 expression (2,3,6,7). Although a solitary fibrous tumour is typically CD34 immunoreactive, CD117 and SMA are generally absent or marginally and focally represented (2,3,6,7). Finally, the differential diagnosis with spindle cell amelanotic melanoma or carcinoma should be performed on the basis of the absence of immunoreactivity for CD117 and CD34 and the appearance of intense staining for melanoma-associated antigens, such as HMB-45, melan-A or cytokeratins.

Predicting the clinical behavior of GISTs remains a complex and noteworthy task, as numerous indicators have been proposed and extensively evaluated without a widespread consensus being achieved (1,3,4,7,12,13,25–29). The most relevant and largely applied morphological parameters have been considered to be tumour size (<5 or >5 cm) and mitotic count (number of mitoses per 50 HPFs) (1,3,4,12,26). Moreover, the site of the GIST has been regarded as a significant predictive aspect. GISTs generally confer a better survival outcome for the patient than tumours of a similar size and mitotic activity occurring in the small intestine, colon and ano-rectum (1,3,4,30). However, the crucial purpose of GIST management is to assess the correct diagnosis in an early phase of the disease in order to realize an adequate curative surgical resection. This is as at a later stage, GISTs may become unresectable or metastatic (5,12,31). In the present study, taking into consideration the aforementioned parameters as guides for evaluating GIST malignancy, it may be concluded that the tumours of the two cases are most likely to be benign, particularly since the maximum diameter of the tumours was <5 cm and ≤3 mitoses were encountered per 50 HPFs. Moreover, the growth fraction of the tumours, as determined by the Ki67 labeling-index, was extremely low, showing <5% of positively-labelled nuclei. However, it has been observed that a certain number of these small and mitotically inactive tumours are later characterized by local recurrence and metastatic disease (1,4,12). Finally, it may be argued that further molecular characterization, for example, the identification of specific KIT mutations that affect various gene domains, may be significant in the selection of tumour subgroups and the prediction of their clinical outcome and response to selective therapy.
